# Impact of Deprescribing Among Older Adults in Primary Care Settings: A Systematic Review and Meta‐Analysis of Randomized Controlled Trials

**DOI:** 10.1002/agm2.70075

**Published:** 2026-04-26

**Authors:** Christina Malini Christopher, Bhuvan KC, Mark Wing Loong Cheong, Rajeev Shrestha, Sunil Shrestha, Juman Dujaili, Ali Blebil

**Affiliations:** ^1^ Discipline of Clinical Pharmacy, School of Pharmaceutical Science Universiti Sains Malaysia Penang Malaysia; ^2^ School of Clinical Sciences Queensland University of Technology Brisbane Australia; ^3^ School of Pharmacy Monash University Malaysia Subang Jaya Selangor Malaysia; ^4^ NIHR Newcastle Patient Safety Research Collaboration Newcastle University Newcastle upon Tyne UK; ^5^ Department of Research and Academics Kathmandu Cancer Center Bhaktapur Nepal; ^6^ Swansea University Medical School Swansea University Singleton Park UK

**Keywords:** deprescribing, medication review, older adults, outcomes, systematic review

## Abstract

**Background:**

Deprescribing is a systematic approach aimed at reducing the number of medications that are no longer beneficial or appropriate. This study aims to assess the various types of deprescribing and the outcomes among older adults in primary health care. The electronic search for original articles was conducted using the following electronic databases: MEDLINE, EMBASE, Scopus, PsycINFO, and Global Health. The types of deprescribing interventions and their outcomes in older adults were assessed and compared with those of non‐intervention. The risk of bias was assessed using the Robins tool. Eighteen randomized controlled trials were included after full‐text screening. These studies evaluated a range of deprescribing interventions implemented in primary healthcare settings. The interventions included medication reviews, academic detailing, pharmacist‐led deprescribing, computerized decision‐support tools, structured education, patient‐centered communication, and coordinated medication risk management. Quantitative analysis revealed that two studies reported a statistically significant reduction in patient falls due to deprescribing (OR: 0.67; 95% CI: [0.46, 0.98]; I^2^ = 0%; *p* = 0.04). Sensitivity analysis further demonstrated that deprescribing significantly reduced the average number of medications used by older adults (MD: 0.17; 95% CI: [0.03, 0.32]; *p* = 0.02) and was associated with a significant decrease in hospitalisations in this population (RR: 1.40; 95% CI: [1.08, 1.80]; *p* = 0.01). Deprescribing interventions in primary care have been proven to result in a modest reduction in the number of medications prescribed to older adults and could help reduce falls, with potential benefits for reducing hospitalization.

**Trial Registration:**

Prospero CRD42023464445

## Graphical abstract

Deprescribing interventions in primary care modestly reduce medication burden, patient falls, and hospitalisations among older adults, highlighting the value of medication reviews and collaborative care strategies.

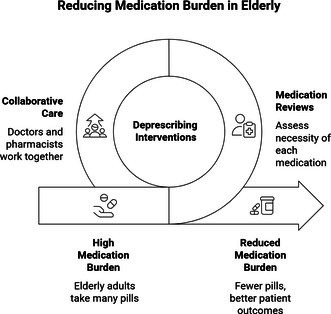

## 1 Introduction

The increasing aging demographic globally has profound implications for the population's medication management. Older populations have a higher prevalence of comorbidities, which often necessitates the use of multiple medications [1]. Older adults frequently exhibit a higher prevalence of comorbidities, necessitating polypharmacy, which affects approximately 40% of global older adults [2, 3]. Polypharmacy results in inappropriate medications and drug contraindications, especially among older adults [4].

Primary care is often the first point of contact where older adults seek treatment. Primary healthcare providers are general practitioners, nurses, pharmacists, physiotherapists, and allied healthcare providers [5]. Various regions reported polypharmacy prevalence in primary care settings: In Portugal, the prevalence of polypharmacy among older adults in primary care practice was 77%, while in Malaysia, it was 50%, and in Qatar, it was 75.5% [6, 7, 8]. Generally, general practitioners and community pharmacists are well‐positioned to communicate on medication initiation, as they can access the patient's full medical history [9, 10]. Studies have shown that support for shared decision‐making and collaborative effort has been the precursor to good prescribing practice among prescribers [11, 12].

Deprescribing has gained substantial attention in the healthcare community in response to these issues. Deprescribing is a systematic approach of reducing or discontinuing no longer appropriate medications or producing beneficial effects to patients [13]. There are various approaches to deprescribing interventions, depending on the settings and healthcare professionals conducting them. Geriatrician or prescriber‐focused deprescribing involves formulating a deprescribing action plan, communicating with pharmacists, and providing health care professionals with the opportunity to proactively identify and reduce inappropriate medications for older adults in hospital settings [14]. Similarly, pharmacist‐focused deprescribing detected inappropriate medication and changes to the pharmacotherapeutic plan [15, 16]. Both of these deprescribing strategies focus on medication regimens among older adults.

Various approaches to deprescribing interventions are being implemented globally. A noteworthy aspect is medication withdrawal, which emphasizes the process of tapering and discontinuing medication use [17, 18]. In addition, medication reviews by a pharmacist were the most frequently practised deprescribing method [19]. Furthermore, computer‐assisted tools designed to identify potentially inappropriate medications have been integrated into medication review processes to facilitate deprescribing [19]. Another finding showed that multidisciplinary collaboration of healthcare professionals, including pharmacists, physicians, and nurses, is recommended as an impactful deprescribing intervention to review and adjust medication regimens [20].

Empirical studies indicate that deprescribing can successfully minimize the prevalence of potentially inappropriate medications and mitigate adverse drug events [21]. Deprescribing results in a reduction in the total number of medications, which directly improves the outcomes of polypharmacy [22]. A recent review report indicates that deprescribing can reverse some risks associated with polypharmacy, focusing on the risk of falling [23]. A randomized controlled trial has demonstrated that a deprescribing intervention reduces the number of medications in the intervention group [24]. A recent review reported that deprescribing interventions are feasible and sufficiently reduce inappropriate medications among hospitalized patients [25]. Engaging in collaborative agreements involving community pharmacists and general practitioners has effectively reduced inappropriate medication use among community‐dwelling older adults [26].

Despite the accumulating evidence of the positive outcomes associated with deprescribing, the challenge of inappropriate polypharmacy among older adults persists as a significant concern [27]. Many patients are often prescribed multiple medications that may not be necessary or beneficial, leading to potential adverse effects and diminished quality of life. Limited evidence exists to compare the effectiveness of various deprescribing interventions in the primary care settings [28]. This gap underscores a need for a more robust emphasis on identifying and implementing effective deprescribing interventions that yield substantial improvements. Therefore, this review aims to evaluate the impact of deprescribing interventions among older adults in primary care settings.

## 2 Methods

The study protocol has been registered at Prospero (CRD42023464445). This review paper was developed based on the Cochrane Handbook for Systematic Reviews of Interventions and the Preferred Reporting Items for Systematic Reviews and Meta‐Analyses (PRISMA) guidelines [29].

### 2.1 Eligibility Criteria

Table provides the detailed population, intervention, comparator and outcomes (PICOS) applied to conceptualize this review. Studies included older adults (over 60 years of age) receiving care in primary care settings. Deprescribing interventions were the main inclusion criteria, and the comparator was either a non‐deprescribing intervention or not receiving deprescribing in primary health care settings. The study design of randomized controlled trials was eligible for this study. Articles published in a language other than English, published before 2013, non‐randomized controlled trials, and no deprescribing intervention were excluded from this review.

### 2.2 Search Strategy

The electronic search for original articles was performed using the following electronic databases: MEDLINE, EMBASE, Scopus, Google Scholar, PsycINFO, Global Health, Cochrane Central Register of Controlled Trials (CENTRAL), and Cochrane Methodology Register for the articles published in the English language from January 1, 2013, to October 30, 2023. Gray literature, such as Google Scholar and the Malaysian Pharmaceutical Society's websites, was also explored to find potential studies relevant to the study objectives and eligibility criteria. The search strategy is explained in Table S2.

### 2.3 Study Selection

Two review authors (CM and MC) screened the titles and abstracts of studies retrieved using the search strategy, as well as those from additional sources, to identify studies that met the inclusion criteria. Data were entered into the MS Excel spreadsheet. Data extraction forms were pilot tested on five studies and revised as needed. Any disagreements were resolved by consensus through another reviewer (AB). Interventions were included if they involved deprescribing interventions in primary care settings, and the study design was a randomized controlled trial.

### 2.4 Data Extraction

A standardized Excel form was used to extract data from studies on design, demographics, setting, interventions, sample size, follow‐up period, outcomes, and main findings. Two authors (CMC and MCW) independently conducted the data extraction, and the third author (AB) resolved any disagreements.

### 2.5 Risk of Bias of Included Studies

The risk of bias of the included studies was assessed by two independent reviewers (CMC and AB). The risk of bias was assessed using the Cochrane Risk of Bias (ROB 2.0) for randomized controlled trials, a revised Cochrane tool [30]. The following characteristics were considered to assess the risk of bias in the included studies: completeness of outcome data and selective outcome reporting. Any disagreements between the review authors over the risk of bias in the included studies were resolved by discussion with the third review author (MCW).

### 2.6 Data Analysis

Studies were eligible for quantitative analysis if the two outcomes were comparable. Statistical heterogeneity was assessed using the I2 statistic, one of the statistical tools for the meta‐analysis study [31]. Based on the reference, heterogeneity was defined as high if I2 > 75% and low if I2 < 25% [32]. The Random effect model was used, assuming heterogeneity exists between studies. All analyses were performed using Cochrane Review Manager version 5.4 (The Nordic Cochrane Centre, Copenhagen, Denmark).

## 3 Results

**FIGURE 1:**
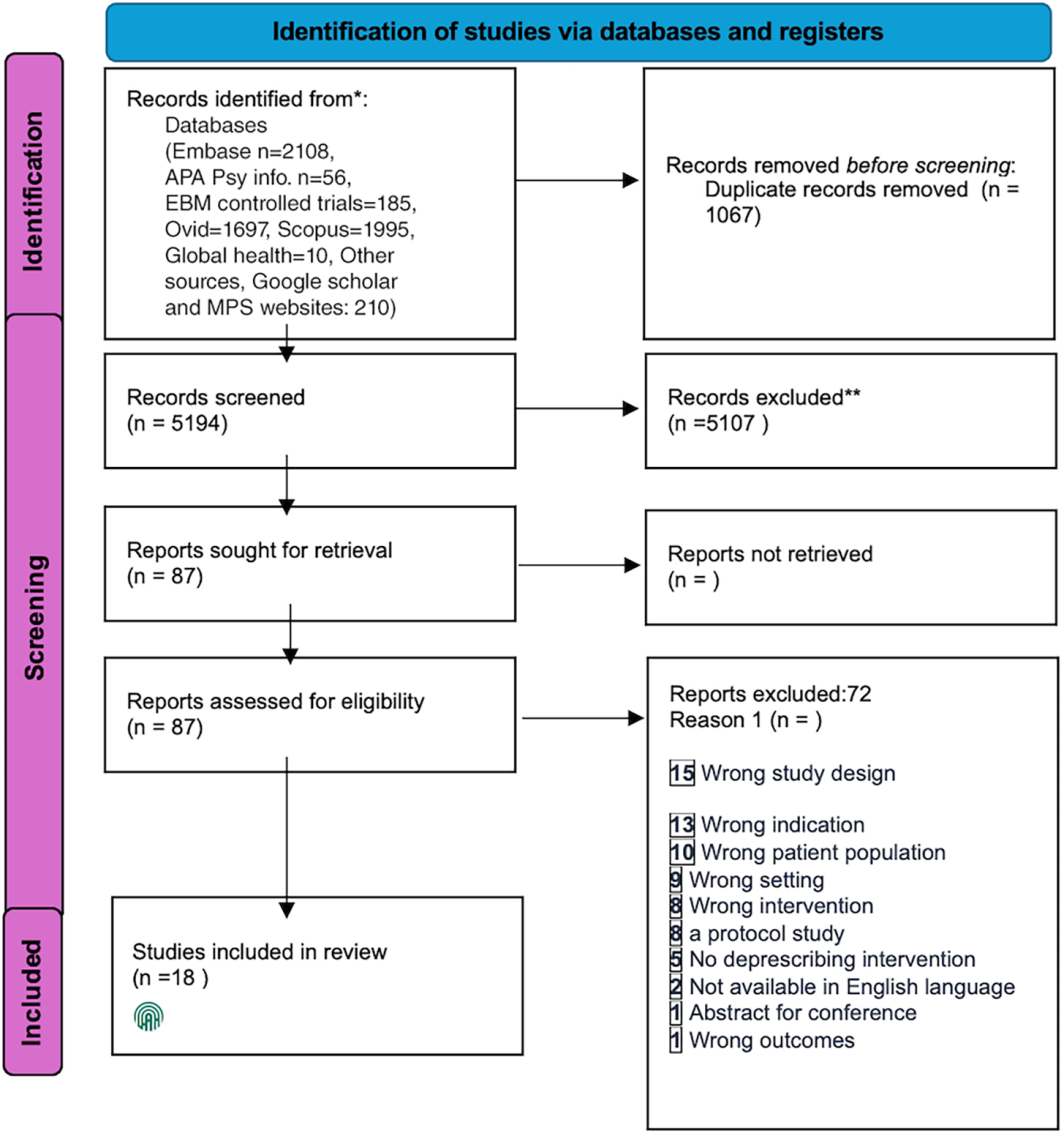
Prisma Flowchart.

The initial search identified 5194 records, and 210 were identified through other methods. A total of 87 full papers were retrieved for further examination. After being screened, 18 studies were extracted, and full texts were assessed for eligibility. The reasons for excluding full texts and the study flow are depicted in Figure 1.

### 3.1 Characteristics of Included Studies

Table 1 shows the summary of the demographics of the included studies. A total of 18 randomized controlled trials were included in this review. Studies were conducted in 12 countries: Australia (two studies) [33, 34] Finland (one study) [35] Canada (two studies) [16, 36] Spain (one study) [37], Switzerland (three studies) [38, 39, 40] Ireland (one study) [41] New Zealand (two studies) [42, 43] Italy (two studies) [44, 45] Germany (four studies) [45, 46, 47, 48] Austria (one study) [45] United Kingdom (one study) [45] and Norway (one study) [49]. The study settings were mainly general practices [33, 37, 40, 41, 45, 47, 49], nursing homes [16, 35, 38] community settings [43] and primary healthcare settings [34, 36, 39, 42, 44, 46, 48]. Deprescribing interventions were conducted through medication review [16, 38, 40, 42, 44], medication review with electronic support, [33, 34, 39, 45, 47] academic detailing, [41, 46, 49] pharmacist‐led deprescribing, [43], computerized decision‐making tool, [36] structured education [37], patient‐centred communication [48], and Coordinated medication risk management [35], (Refer to Table S3 for full study characteristics).

**TABLE 1:** Summary of demographics.

Variable	Number of studies (*n* = 18)
Duration of study	
3–6 months	6 (33.3%)
6–12 months	8 (44.4%)
> 1 year	4 (22.2%)
Country	
Australia	2 (11.1%)
Canada	2 (11.1%)
Switzerland	3 (16.7%)
Ireland	1 (5.5%)
New Zealand	2 (11.1%)
Italy	2 (11.1%)
Germany	4 (22.2%)
Austria	1 (5.5%)
United Kingdom	1 (5.5%)
Norway	1 (5.5%)
Finland	1 (5.5%)
Spain	1 (5.5%)
Approach of deprescribing intervention	
Medication review	6 (33.3%)
Medication review with electronic support	5 (27.8%)
Academic detailing	1 (5.5%)
Pharmacist‐direct led	1 (5.5%)
Computer decision making tool	1 (5.5%)
Patient centered communication	1 (5.5%)
Structured Education to prescribers	2 (11.1%)
Coordinated medication risk management	1 (5.5%)
Family conferences	1 (5.5%)
Intervention provider	
Doctors	15 (83.3%)
Pharmacists	8 (53.3%)
Nurses	2 (11.1%)
Outcomes	
Hospitalization	5 (27.8%)
Mortality	6 (33.3%)
Number of patient falls	2 (11.1%)
Mean number of medications	9 (50%)
Potentially inappropriate medications	6 (33.3%)
Drug burden index	3 (16.7%)
Medication appropriateness index	2 (11.1%)
Quality of life	5 (27.8%)

### 3.2 Risk of Bias

Figure S1 illustrates the risk of bias for all included studies in each domain. Overall, six studies were judged to have a low risk of bias, ten studies were judged to have some concerns regarding the level of risk, and two studies were found to have a high risk of bias. Two studies showed a high risk of bias in the randomization process [33, 43]. Only one study showed a high risk of bias in selecting reported results [43].

### 3.3 Deprescribing Interventions

#### 3.3.1 Prescriber‐Led Medication Review

Prescriber‐led medication review is an intervention that focuses on the initiative of prescribers to examine medication indications among patients. These were conducted based on the discussion of initiation and reduction of medications between GPs and patients [33]. Prescribers reviewed medications and conducted further consultations and referrals. Pharmacists also conducted deprescribing by allowing pharmacological and nonpharmacological management modification in collaboration with prescribers [38]. Most of the medication reviews with the training were focused on deprescribing with the incorporation of deprescribing workshops, information, and discussion for prescribers [33]. Another intervention on patient‐centred communication directed at deprescribing was the general practitioner‐led intervention [48]. General practitioners (GPs) conducted extra consultations with each patient in addition to routine appointments. The first consultation focused on identifying the patient's treatment goals and priorities. The second consultation involved a discussion of the patient's medication regimen, utilizing a “brown bag” review of all medications the patient had at home. The third consultation addressed achieving treatment goals and planning for future therapeutic targets. However, this intervention did not successfully lead to a reduction in the number of medications.

#### 3.3.2 Medication Review With Electronic Support

This intervention is defined as reviewing medication using electronic health records (EHRs), clinical decision support systems (CDSS), or other digital technologies. This intervention differs from medication review because it includes a web‐based electronic clinical decision support system based on the STOPP/START criteria version [39]. Kouladjian O'Donnell et al. [34] carried out a deprescribing intervention using a computerized clinical decision support system to support person‐centred medication reviews. Although the decision support tool was designed to promote the deprescribing of potentially inappropriate medications, the ultimate decisions regarding treatment remained under the discretion of both the physician and the patient within the framework of shared decision‐making [45].

#### 3.3.3 Academic Detailing

This intervention provides a concept of, and educational outreach strategy aimed at improving prescribing practices, specifically to promote the safe and appropriate discontinuation of medications among older adults. Academic detailing for deprescribing is an intervention of prescribers' practice against some recommended deprescribing initiatives to improve the prescribing pattern [49]. Academic detailing could compromise educational modalities such as clinical case discussion and practical deprescribing tools to address prescribing. The multifaceted intervention involved academic detailing with a pharmacist on how prescribers can review medicines with participating patients; the medicine reviews were supported by web‐based pharmaceutical treatment algorithms for prescribers that provided evidence‐based alternative treatment options [41]. Mortsiefer et al. [46] studied with approaching pharmacists to assess how prescribers review medicines with participating patients, and medication is supported by web‐based pharmaceutical treatment algorithms [46].

#### 3.3.4 Pharmacist‐Led Deprescribing

Pharmacist‐led deprescribing is coordinated or supported by a pharmacist to identify, reduce, or discontinue medications. One study conducted direct pharmacist‐led deprescribing, an immediate discussion with the patient, caregivers, or family members regarding their medications [43] It was conducted in patients' homes. After the discussion, the pharmacist recommended that patients' prescribers possibly deprescribe sedative and anticholinergic medicines to reduce inappropriate medications.

#### 3.3.5 Other Intervention

Another study implemented a computerized decision‐support tool as a part of a deprescribing initiative, focusing solely on electronic assistance [36]. Prescribers received alerts detailing the nature of the issue, its potential consequences, and suggested alternative treatments. In a separate intervention, structured education was provided to prescribers through either follow‐up visits (Structured Intervention with Follow‐up, SIF) or written instructions (Structured Intervention with Written instructions, SIW) [37]. This approach offered targeted recommendations for managing long‐term benzodiazepine use.

### 3.4 Outcomes

#### 3.4.1 Mean Number of Medications

Four studies examined the effect of deprescribing intervention on the mean number of medications at the end of the study [34, 35, 45, 48]. The quantitative analysis showed that these three pooled studies did not show a statistically significant impact of medication review on the probability of the number of medications (MD 0.07; 95% CI [−0.14, 0.28] *I* ^*2*^ = 81%, *p* = 0.53, Figure S2).

#### 3.4.2 Number of Hospitalized Patients

Four studies reported the number of hospitalized patients as the outcome of the intervention [38, 44, 45, 47]. The pooled analysis showed that these studies did not show a statistically significant impact of deprescribing intervention on the probability of hospitalization (MD 1.04; 95% CI [0.79, 1.37] I^2^ = 70%, *p* = 0.79, Figure S3).

#### 3.4.3 Number of Mortalities

Three studies explored the outcomes of deprescribing interventions on the number of mortalities [38, 44, 45]. The quantitative analysis showed that these three pooled studies did not show a statistically significant impact of deprescribing on the probability of mortality (OR 1.07; 95% CI [0.92, 1.25] *I* ^*2*^ = 0%, *p* = 0.40, Figure S4).

#### 3.4.4 Number of Patient Fall

**FIGURE 2:**
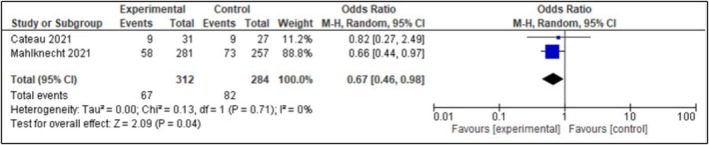
Number of patient falls.

Two studies were analyzed quantitatively to examine the effects of deprescribing on the number of patient falls [38, 44]. The quantitative analysis showed that these two studies showed a statistically significant impact of deprescribing on the number of patient falls (OR 0.67; 95% CI [0.46, 0.98] *I* ^*2*^ = 0%, *p* = 0.04, Figure 2).

#### 3.4.5 Sensitivity Analysis

**FIGURE 3:**
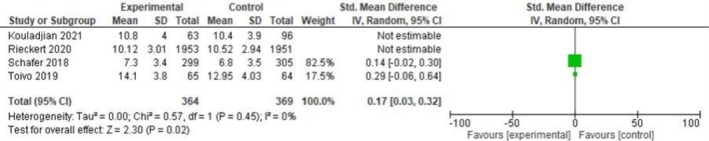
Sensitivity analysis on the number of medications.

High heterogeneity, *I* ^*2*^ = 81%, was found in the outcome of the mean number of medications. Thus, a one‐on‐one removal of studies in the meta‐analysis was done by removing two studies: Kouladjian O'Donnell et al. [34] and Rieckert et al. [45] in the mean number of medication outcome, and no heterogeneity was found. This analysis reported that deprescribing was significant in the mean number of medications among older adults (MD 0.17; 95% CI [0.03,0.32] *I* ^*2*^ = 0%, *p* = 0.02, Figure 3).

**FIGURE 4:**
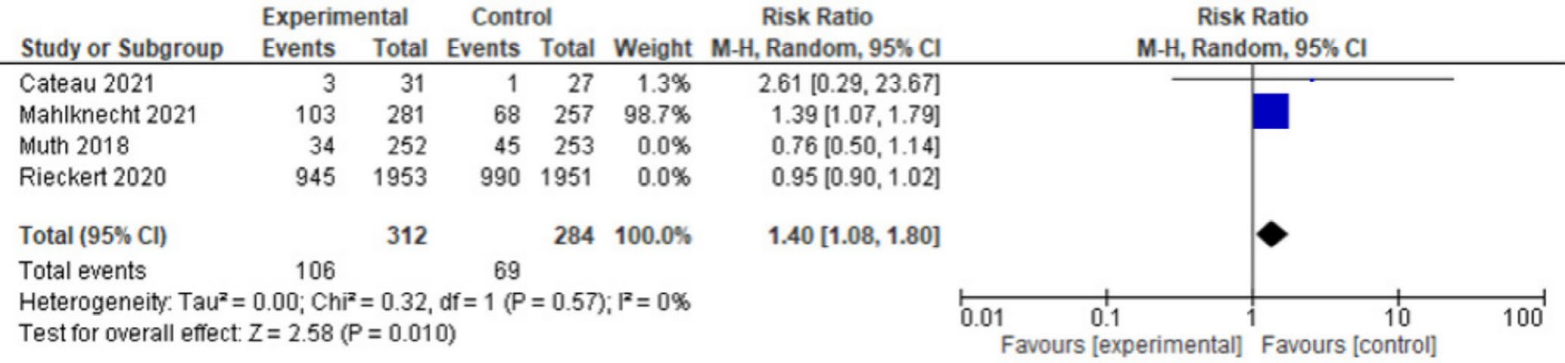
Sensitivity analysis on hospitalization.

Two studies were removed from the hospitalization outcome, which also showed high heterogeneity of 70%. Muth et.al. [47] and Rieckert et.al. [45] were conducted. The analysis showed that deprescribing was significant in reducing the number of hospitalized older adult patients (RR 1.40; 95% CI [1.08,1.80] *I* ^*2*^ = 0%, *p* = 0.01, Figure 4).

#### 3.4.6 Potentially Inappropriate Medications

One study showed evidence that the deprescribing intervention significantly benefited the intervention group, which had lower odds of receiving potentially inappropriate prescriptions compared to the control group [41]. Similarly, Mortsiefer et al. [46] found that the number of potentially inappropriate medications was significantly lower in the intervention group (mean 1.30 [SD 1.05]) than in the control group (mean 1.71 [SD 1.25]; *p* = 0.04). Tamblyn et al. [36] also reported an 18% reduction in new potentially inappropriate prescriptions per 1000 visits in the deprescribing group. However, two studies found no significant effect of the intervention on reducing inappropriate medications [35, 38].

#### 3.4.7 Drug Burden Index

Two studies showed that pharmacist‐led medication review, a deprescribing intervention, did not reduce the anticholinergic/sedative load within 6 months [34, 42]. The only difference between these studies was medication review with and without electric support. On the contrary, Vicens et al. [37] reported that deprescribing interventions focusing on prescribers' education on benzodiazepine reduction doses were effective in reducing benzodiazepine use after 3 years.

#### 3.4.8 Other Outcomes

Zechmann et al. [40] and Schäfer et al. [48] provided evidence that medication review and narrative medicine‐based deprescribing interventions did not impact quality of life outcomes. Similarly, Jungo et al. [39] reported that their medication review with electronic support intervention failed to improve the patient's quality of life. One study reported improvement in medication appropriateness (OR 1.05, 95% CI [0.59, 1.87]) [39]. However, the medication review, prioritizing multiple medications in multimorbidity, did not impact the medication appropriateness index [47].

## 4 Discussion

To the best of our knowledge, this systematic review and meta‐analysis is the first to examine both the types of deprescribing interventions commonly used and their impact on health outcomes among older adults in primary healthcare settings. Our review differed from Persaud et al. [50] in that it focused on older adults living in the community or in long‐term care facilities, and our review specifically concentrated on primary healthcare facilities. Medication review was the most frequently implemented among other various approaches, highlighting the importance of interprofessional collaboration in deprescribing practices. Quantitative findings indicate that medication reviews can reduce the number of patient falls. Additionally, sensitivity analyses suggest that deprescribing positively reduces hospitalisations and the average number of medications used.

Previous reviews have shown that deprescribing has reduced the number of medications [23, 28]. Following that, findings align with Omuya et al. [51] which found a significant deprescribing impact on HRQOL, cost, or hospitalization. Our findings aligned with Kua et al. [52] that deprescribing interventions significantly reduced the number of older adults' medications. Mixed results were found for inappropriate prescriptions and QoL due to intervention variability and follow‐up duration.

As for mortality, our findings failed to provide a significant reduction among older adults with the deprescribing intervention. In contrast, another review's finding claimed that by further subgroup analyses of randomized studies on deprescribing polypharmacy, a significant reduction in mortality [53]. In other findings, the number of falls was not a consistently significant outcome, which conflicts with the current review [54].

Deprescribing intervention could be conducted using several approaches, as analyzed in this review. Medication reviews and medication reviews with electronic support were favorable among prescribers and pharmacists [55, 56, 57]. The intervention is similar and differs in terms of using the guidance of software or online tools to detect inappropriate medications. Other approaches of intervention in this current review, such as patient‐centred approaches and coordinated risk management, have shown a significant reduction in the number of medications. Similarly, medication review only supports the reduction of the number of hospitalisations among older adults. Therefore, various deprescribing interventions could be focused on addressing the impact on medication use and other health outcomes. Deprescribing intervention is widely practised in developed countries, as assessed in this review. However, there are limited studies conducted in lower‐ and middle‐income countries. From this review, suggestions could be made to provide more comprehensive frameworks in lower‐middle‐income countries to enhance medication use and health outcomes among the older population in primary healthcare.

### 4.1 Strengths and Limitations

Firstly, this review has included only randomized controlled trials, increasing the study's robustness. Heterogeneity across studies was also assessed with sensitivity analysis, and we have reported the homogeneity of studies after removing one study. Additionally, this current review included all studies across the healthcare settings globally, searching several databases and supplemented by a manual search and a search in Google Scholar to include all potential studies.

However, there are a few limitations. Firstly, the articles in the search for this review were restricted to English. Thus, we acknowledge that the search might be limited to non‐English native regions. In addition, the study couldn't analyze pooled estimates for some outcomes because the studies were too different in what they measured or how they measured it.

### 4.2 Implication for Practice and Future Recommendations

This review offers potential strategies to enhance collaboration among healthcare providers, including prescribers, pharmacists, nurses, and older adult patients, as well as their caregivers, in understanding and implementing medication deprescribing. Effectively applying these strategies can support more personalized care, improve patient outcomes, and promote a culture of shared decision‐making within healthcare systems. Implementation is especially important for addressing the challenges of polypharmacy and ensuring that treatment plans are individualized, balancing the complexity of medication regimens with the goal of maximizing therapeutic benefits. Future research should aim to identify the most effective deprescribing strategies across various healthcare settings and examine the long‐term impact of these interventions on the health and well‐being of older adults.

## 5 Conclusion

This review provides evidence that medication review is the most used deprescribing intervention approach in primary healthcare and shows promising results in reducing medication burden and hospitalisations. However, the effects on other outcomes, such as adverse drug events, quality of life, and functional status, remain mixed and require further investigation. Deprescribing strategies should be individualized to meet the specific needs of older adults, considering their comorbidities, preferences, and overall treatment goals. Further research is needed to optimize these interventions and evaluate their long‐term impact on patient health and well‐being.

## Author Contributions

C.M.C.: conceptualisation, methodology, formal analysis, writing original draft. B.K.C.: methodology, writing original draft. M.W.L.C.: data curation, methodology, writing original draft, supervision. R.S.: revision of original draft. S.S.: methodology, revision of original draft. J.D.: data curation, methodology, revision of original draft. A.B.: data curation, methodology, revision of original draft, supervision.

## Funding

The authors have nothing to report.

## Consent

The authors have nothing to report.

## Conflicts of Interest

The authors declare no conflicts of interest.

## Supporting information


**Table S1:** Population, Intervention, Comparator, Outcomes, and Study design (PICOS).
**Table S2:** Search strategy.
**Table S3:** Table of characteristics.
**Figure S1:** Risk of bias.
**Figure S2:** Mean number of medications.
**Figure S3:** Number of hospitalized patients.
**Figure S4:** Number of mortality.

## Data Availability

All data supporting the findings of this study are available within the paper and its Supporting Information.
