# Supplementary material for: Impact of Deprescribing Among Older Adults in Primary Care Settings: A Systematic Review and Meta‐Analysis of Randomized Controlled Trials
Source: Aging Med (Milton). 2026 Apr 26;9(2):214–23. doi: 10.1002/agm2.70075 (PMC13163933; doi:10.1002/agm2.70075)
Supplement: Supplementary file 1 — Table S1: Population, Intervention, Comparator, Outcomes, and Study design (PICOS). Table S2: Search strategy. Table S3: Table of characteristics. Figure S1: Risk of bias. Figure S2: Mean number of medications. Figure S3: Number of hospitalized patients. Figure S4: Number of mortality. [file AGM2-9--s001.docx]

**Appendix**

Appendix 1 Population, Intervention, Comparator, Outcomes, and Study design (PICOS)

| Population | Older adults aged 60 or above receiving care in community-based primary care settings, including general practices, community pharmacies, and outpatient primary care clinics |
| --- | --- |
| Intervention | Deprescribing |
| Comparator | Non deprescribing intervention |
| Outcomes | Any health outcomes |
| Study design | Randomized controlled trial |

Appendix 2

Search strategy

| 1. | Middle Aged/ or Aged/ or Aging/ or "older adults".mp. or "Aged, 80 and over"/ |
| --- | --- |
| 2. | geriatric.mp. or Geriatrics/ |
| 3. | elderly.mp. or Aged/ |
| 4. | Aging/ or aging.mp. |
| 5. | ageing.mp. |
| 6. | older.mp. |
| 7. | veteran.mp. or Veterans/ |
| 8. | "60 years".mp. |
| 9. | Deprescrib*.mp. |
| 10. | Inappropriate Prescribing/ or Inappropriate.mp. |
| 11. | reduc*.mp. |
| 12. | stop*.mp. |
| 13. | withdraw*.mp. |
| 14. | cessation.mp. |
| 15. | discontinu*.mp. |
| 16. | cessation.mp. |
| 17. | "health outcomes".mp. |
| 18. | falls.mp. |
| 19. | Hospitalization/ or hospitali*.mp. |
| 20. | Mortality/ or mortality.mp. |
| 21. | "medication adherence".mp. or Patient Compliance/ or Medication Adherence/ |
| 22. | "medication compliance".mp. or Medication Adherence/ |
| 23. | polypharmacy.mp. or Polypharmacy/ |
| 24. | "quality of life".mp. or "Quality of Life"/ |
| 25. | cognitive.mp. |
| 26. | "Drug-Related Side Effects and Adverse Reactions"/ or "medication appropriateness".mp. or Pharmacists/ |
| 27. | "Adverse drug event".mp. or "Drug-Related Side Effects and Adverse Reactions"/ |
| 28. | Medication Errors/ or "medication administration".mp. |
| 29. | satisfaction.mp. |
| 30. | 1 or 2 or 3 or 4 or 5 or 6 or 7 or 8 |
| 31. | 9 or 10 or 11 or 12 or 13 or 14 or 15 or 16 |
| 32. | 17 or 18 or 19 or 20 or 21 or 22 or 23 or 24 or 25 or 26 or 27 or 28 or 29 |
| 33. | Primary Health Care/ or "primary healthcare".mp. |
| 34. | Community Pharmacy Services/ or Pharmacies/ or "community pharmac*".mp. or Pharmacists/ |
| 35. | clinic*.mp. |
| 36. | "general practitioners".mp. or General Practitioners/ |
| 37. | 33 or 34 or 35 or 36 |
| 38. | 30 and 31 and 32 and 37 |
| 39. | limit 38 to (english language and "all aged (65 and over)" and english) |

Appendix 3

Table of characteristics

| **No** | **Authors, Year Country** | **Settings,** | **Type of Intervention** | **Intervention provider** | **Sample size** | **Follow-up period** | **Outcomes** | **Findings** | **Conclusion** |
| --- | --- | --- | --- | --- | --- | --- | --- | --- | --- |
| 1 | Anderson et al. (2020)  Australia | Five general practice clinics,  Exploratory pragmatic clinical trial | Medication review using electronic software template (CMR) | General practitioners and pharmacists  at theGP’s discretion. | I: 120  C: 101 | 18 weeks | Primary: Mean difference in number of regular medicines  Secondary: Medicine-specific and patient-reported outcome | - Mean (SD) number of regular  medicines deprescribed per patient was 0.99 (1.23) in the inter-  vention group vs 0.43 (0.84) in the usual care group, equaling a  mean difference of 0.55  - Therewere no statistically significant in secondary outcomes. | The deprescribing intervention was found to be feasible and modestly effective in reducing potentially inappropriate polypharmacy in older adults |
| 2 | (Balsom et al., 2020)  Canada | A long term care facility  Parallel design RCT | Medication review  The intervention involved pharmacy students conducting medication reviews under pharmacist supervision to identify medications for deprescribing .  - Participants received a comprehensive medication review, focusing on potentially harmful medications . | Pharmacist | I: 22  C: 23 | 3 and 6 months | Change in the number of medication | -The mean number of medications in the intervention  group was 2.68 less than the control group (p < 0.02; 95% CI − 4.284, − 1.071) at 3 months and 2.88 less (p = 0.02, 95% CI  − 4.543, − 1.112) at 6 months. | A pharmacist-led deprescribing intervention can reduce the number of unnecessary and  potentially harmful medications |
| 3 | (Cateau et al., 2021)  Switzerland | Nursing homes  Controlled clinical trial | Medication review  Pharmacist-led, deprescribing-focused medication review in collaboration with nurses and physicians. | Pharmacist, nurses and physicians | I:31  C: 27 | 4 months | Primary: Number and dose of PIMs  Secondary: safety outcomes (mortality, hospitalisations, falls, and use of physical restraints). | The pharmacists proposed 169 modifications, and 49% were accepted and implemented. Defined Daily Doses (DDD) significantly reduced in the intervention group (IRR 0.763, 95% CI [0.594, 0.979]), with a more marked effect at chronic drugs, with a 28% reduction (IRR 0.716, 95% CI [0.546, 0.938]) in the number of long-term PIM DDDs.  - No significant changes were seen on mortality, hospitalisations, falls, and  restraints use, | The deprescribing intervention did not significantly reduce the number of potentially inappropriate medications (PIMs) used by participants .  - There was a potential benefit in reducing the doses of PIMs used .  - Some participants experienced adverse consequences, potentially affecting their quality of life . |
| 4 | (Clyne et al., 2015)  Ireland | General practice clinics  Cluster-randomized controlled trial | Academic detailing  Medication review with web-based pharmaceutical treatment algorithms | Pharmacists and General practitioners | I: 99  C: 97 | 4-6 months | Primary: Proportion of patients  with PIP  Secondary: The mean number of potentially inappropriate prescriptions. | Intervention group had significantly lower odds of having PIP  than patients in the control group (adjusted odds ratio = 0.32; 95% CI, 0.15-0.70;  P = .02)  The mean number of PIP drugs in the intervention group was 0.70, com-  pared with 1.18 in the control group (P = .02). T | Academic detailing was effective in reducing PIP by deprescribing inappropriate medications. |
| 5 | (Jamieson et al., 2023)  New Zealand | Two district health boards | Medication review | Pharmacist | I:184  C:179 | 6 months | Primary:Drug burden index,  Secondary: emergency department (ED) visits, hospital admissions,  admissions to ARC, and mortality. | -21 (12.7%) in the control group and 21 (12.2%) in the intervention group had a reduction in DBI ≥ 0.5.  -The hazard ratio (HR) for  presentation to ED was 1.06 (95% CI: 0.82 to 1.39)  -The HR for  hospital admission was 1.24 (95% CI: 0.74 to 2.06).  -The HR for death was 0.98 (95% CI: 0.45  to 2.10) | Pharmacist-led medication review of frail older participants did not reduce the anticholinergic/sedative load within 6 months |
| 6 | (Jungo et al., 2023)  Switzerland | Primary care settings | Medication  review intervention centred around an electronic  clinical decision support system (eCDSS) | General practitioners | I:160  C:163 | 12 months | Medication Appropriateness Index (MAI)  Secondary: Number of medications falls, fractures, and quality of life. | The improvement in appropriateness of medication  (odds ratio 1.05, 95% confidence interval 0.59 to  1.87) and the number of prescribing omissions (0.90,  0.41 to 1.96) were inconclusive.  -No significant improvement in secondary outcomes | The results revealed that this intervention effectively reduced inappropriate prescriptions, minimized polypharmacy, and improved the quality of prescribing for older adults with multiple health conditions |
| 7 | (Kouladjian O'Donnell et al., 2021)  Australia | Primary care clinics | Medication Review Electronic Decision Support System  (G-MEDSS) | Pharmacists and general practitioners | I:88  C: 113 | 3 months | Primary: Proportion of patients with any reduction in  DBI  Secondary: HMR recommendations to change DBI a | -The proportion of patients with a reduction in DBI was not significantly differ-  ent at 3-months (intervention 17%, comparison 11%; adjusted odds ratio 1.44, 95%  confidence interval 0.56–3.80).  - the HMR report made recommendations to reduce DBI for a significantly greater proportion of patients in the  intervention than in the comparison group (intervention 37%, comparison 14%;  adjusted odds ratio 3.20, 95% confidence interval 1.50–6.90). | Implementation of G-MEDSS within HMR did not reduce patients' DBI  at 3 months compared with usual care HMR. |
| 8 | (Mahlknecht et al., 2021)  Italy | Primary care clinics | Medication review | General practitioners | I:281  C:257 | 24 months | Primary: Hospitalisation  Mortality  Secondary: Number of medications, falls, fractures, quality of life, affective  status, cognitive function. | -The adjusted rates of  occurrence of the primary outcome in the CG and IG  groups did not differ significantly (ITT: adjusted OR 1.46,  95%CI 0.99–2.18, p = 0.06; PP: adjusted OR 1.33, 95%CI  0.87–2.04, p = 0.2)  -Reduction in fall favouring the IG was observed in the adjusted  significant  analysis (adjusted OR 0.55, 95%CI 0.31–0.98; p = 0.04).  No statistical significant on other outcomes | A modest reduction in inappropriate medications can yield clinical benefits without negatively impacting patient-related outcomes .  - Significant reductions in falls were observed, despite limited discontinuation of inappropriate medications .  - The implementation rate of deprescribing suggestions was low, indicating a need for better training for GPs |
| 9 | (Mortsiefer et al., 2023)  Germany | Primary care clinics | Family conferences | General practitioners (GPs) | I: 272  C: 249 | 12 months | Primary: Number of hospitalizations  Secondary: Number of medications  Number of  potentially inappropriate medication (EU[7]-PIM) | -No significant difference  in the adjusted mean (SD) number of hospitalizations between the intervention group (0.98 [1.72])  and the control group (0.99 [1.53])  - the mean  (SD) number of medications decreased from8.98(3.56) to8.11 (3.21) at6months and to8.49(3.63)  at 12 months in the intervention group  - the mean (SD) number of  EU(7)-PIMswas significantly lower in the intervention group (1.30 [1.05]) than in the control group  (1.71 [1.25]; P = .04). | The study found no significant difference in hospitalizations between intervention and control groups over 12 months .  - Family conferences initiated the process of deprescribing but did not yield clinical benefits in hospitalization rates .  - The reduction in potentially inappropriate medications was significant at 6 months but not retained at 12 months . |
| 10 | (Muth et al., 2018)  Germany | General practice clinics | Medication review  (PRIMUM) intervention  A computerized decision support system (CDSS) assisted the general practitioner (GP) in optimizing medication during consultations. | General practitioner | I: 252  C: 253 | 9 months | Primary: Medication Appropriateness Index  Secondary: quality of life, functioning, medication  adherence | -MAI sum scores decreased by 0.3 points in IG and  0.8 points in CG, resulting in a non-significant adjusted  mean difference of 0.7 (95% CI −0.2 to 1.6) points in  favour of CG.  - Non-significant  Changes for secondary outcomes | The study found that the inclusion of family in discussions and decision-making regarding medication reduction led to more informed and successful deprescribing outcomes. |
| 11 | (Nishtala et al., 2023)  New Zealand | Community settings  Pragmatic cluster randomised controlled trial | Pharmacist-led deprescribing | Pharmacist-led deprescribing were conducted at participants’ home.Pharmacist then sent a letter to their physician/general  practitioner (GP) (family doctor) to consider possible deprescribing of  sedative and anticholinergic medicines where appropriate. | I:112  C:111 | 6 months | Anticholinergic burden (ACB) | No significant impact.The mean change in ACB for those in the control arm was 0.19, and the intervention arm  was 0.23. | The study found no meaningful evidence supporting the pharmacist deprescribing intervention's effectiveness in reducing anticholinergic burden . |
| 12 | (Rieckert et al., 2020)  Austria, Germany, Italy, and  the United Kingdom. | General practice clinics,  Pragmatic multicentre, cluster randomised controlled  trial. | Electronic decision support tool | Doctors received training and instructions  on use, interpretation of the output, the evidence base  underpinning the output, and the principles of shared  decision making. They then implement deprescribing based on the recommendations. | I:1953  C:1951 | 24 months | Primary: Hospitalisation or mortality  Seondary: Number of medications | Hospitalisation or death occured 871  (44.6%) participants in the intervention group and  944 (48.4%) in the control group.  - The number of prescribed drugs had  decreased in the intervention group compared with  control group (uncontrolled mean change −0.42  v 0.06: adjusted mean difference −0.45, 95%  confidence interval −0.63 to −0.26; P<0.001 | A computerised decision  support tool for comprehensive drug review of elderly  people with polypharmacy showed reduction in medications. |
| 13 | (Rognstad et al., 2013)  Norway | General practice clinics  Cluster randomised controlled trials | Multifaceted education for GP | Peer academic detailers provided two 2-day pre-study training sessions,  focusing on: safety issues in relation to  pharmacological treatment in older people;  the rationale for the 13 listed PIPs;  and how to facilitate learning within a  group setting. | I:256  C: 209 | 12 months | Changes  in prescription patterns a | PIPs was reduced by 3.3%  or 12.1% relative to baseline | The study's multifaceted educational intervention led to a 12.1% reduction in potentially inappropriate prescriptions (PIPs) for older patients . |
| 14 | (Schäfer et al., 2018)  Germany | 55 primary care practices  Two-arm cluster-randomised controlled tria | Narrative medicine-based intervention | GP identifys treatment targets and priorities of  the patient and discuss goal attainment and future  treatment targets | I: 325  C: 325 | 12 months | Number of medications and health related quality of life | There was no difference between treatment  and control group in the change of the number of  medications taken (0.43; 95% CI −0.07 to 0.93; P=0.094)  and no difference in health-related quality of life (0.03;  −0.02 to 0.08; P=0.20 | This study shows e effects of a multifaceted intervention based on  the Chronic Care Model and Narrative Based Medicine  on the number of medications taken and health-related  quality of life |
| 15 | (Tamblyn et al., 2003)  Canada | Primary care setting  Cluster-randomized controlled trial | Computerized decision-making (MOXXI) | The intervention involves integrating this computerized system into primary care practices to support healthcare providers in improving their prescribing practices and reducing instances of inappropriate medication prescriptions for patients. | I: 54  C:53 | 13 months | The rate of initiation of  potentially inappropriate prescriptions | The number of new potentially inappropriate pre-  scriptions per 1000 visits was significantly lower (18%) in the  CDS group than in the control group (relative rate [RR] 0.82,  95% confidence interval [CI] 0.69–0.98 | This study supports healthcare providers to make more informed decisions and enhance patient safety by minimizing inappropriate prescribing practices. |
| 16 | (Toivo et al., 2019)  Finland | 5 home care units, the public healthcare  center, and a private community pharmacy  Cluster randomized controlled trial | Coordinated medication risk  management | Multidisciplinary team | I: 65  C:64 | 12 months | -PIM  -Mortality | The intervention did not show an impact on the medication risks between the original  intervention group and the control group in the intention to treat analysis, but the per protocol analysis indicated  tendency for effectiveness, particularly in optimizing central nervous system medication use | The care coordination intervention used in this study indicated  tendency for effectiveness when implemented as  planned, particularly in optimizing CNS medication use  during a 12-month follow-up. |
| 17 | (Vicens et al., 2016)  Spain | Three-arm multicentre, cluster randomised trial | General practitioner’s information | General practitioners | Ia:191  Ib:168  C: 173 | 36 months | Benzodiazepine use | At 36 months, 66/168 patients (39.2%) in the  SIW group, 79/191 patients (41.3%) in the  SIF group, and 45/173 patients (26.0%) in the  control group had discontinued BZD use. | The interventions were effective on cessation  of BZD use; most patients who discontinued  at 12 months remained abstinent at 3 years. |
| 18 | (Zechmann et al., 2020)  Switzerland | General practice clinics  cluster-randomised clinical study | Medication review | Primary care physicians | I:128  C:208 | 12 months | -Mean difference in the number of drugs per patient  -Patient safety  -Quality of life | The intervention group experienced a significant immediate reduction in the number of drugs per patient right after the intervention, with 81% of the drugs stopped during the consultation remaining discontinued after 12 months.  There were no significant differences in clinical events, hospitalizations, death rates, or quality of life (QoL) measures between the intervention and control groups at any time point throughout the study | Patient-centred deprescribing procedure is effective immediately after the  intervention, but not after 6 and 12 months |

Appendix 4


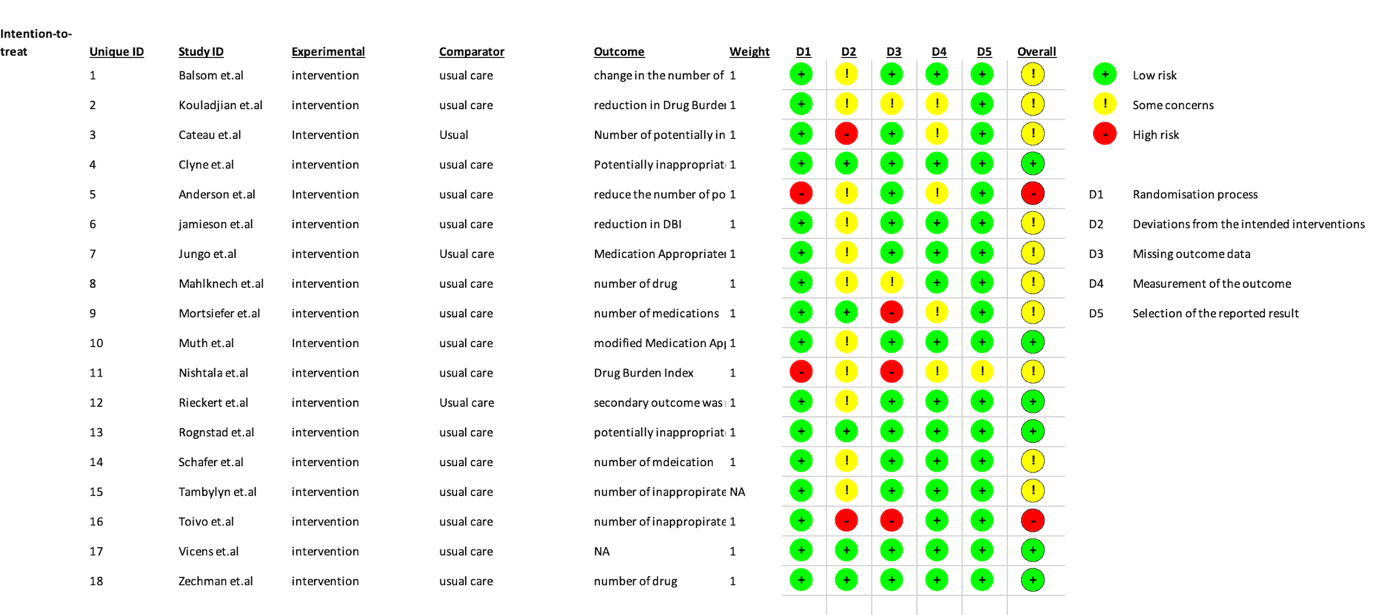


Figure 1 Risk of bias

Appendix 5


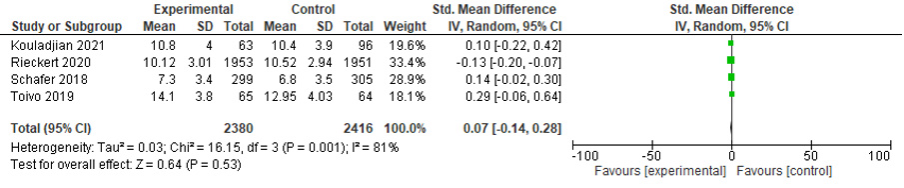


Figure 2 Mean number of medications

Appendix 6


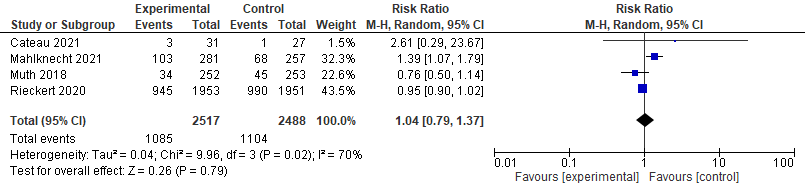


Figure 3 Number of hospitalized patients

Appendix 7


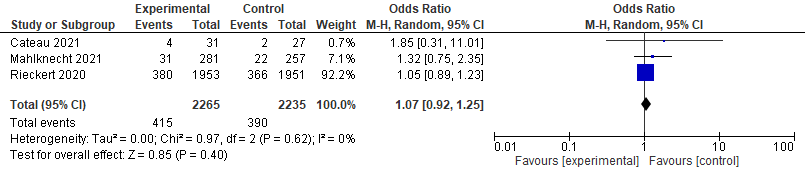


Figure 4 Number of mortality
